# Prognosis of the non-ST elevation myocardial infarction complicated with early ventricular fibrillation at higher age

**DOI:** 10.1007/s11357-021-00377-3

**Published:** 2021-05-14

**Authors:** Réka Skoda, György Bárczi, Hajnalka Vágó, Attila Nemes, Liliána Szabó, Gábor Fülöp, István Hizoh, Dominika Domokos, Klára Törő, Elek Dinya, Béla Merkely, Dávid Becker

**Affiliations:** 1 Heart and Vascular Center, Semmelweis University, Városmajor u. 68, Budapest, 1122 Hungary; 2 Department of Medicine, Albert Szent-Györgyi Clinical Center, Medical Faculty, University of Szeged, Szeged, Hungary

**Keywords:** Early ventricular fibrillation, NSTEMI, Prognosis, Aging

## Abstract

Early ventricular fibrillation (EVF) predicts mortality in ST-segment elevation myocardial infarction (STEMI) patients. Data are lacking about prognosis and management of non-ST-segment elevation myocardial infarction (NSTEMI) EMI with EVF, especially at higher age. In the daily clinical practice, there is no clear prognosis of patients surviving EVF. The present study aimed to investigate the risk factors and factors influencing the prognosis of NSTEMI patients surviving EVF, especially at higher age. Clinical data, including 30-day and 1-year mortality of 6179 NSTEMI patients, were examined; 2.44% (*n*=151) survived EVF and were further analyzed using chi-square test and uni- and multivariate analyses. Patients were divided into two age groups below and above the age of 70 years. Survival time was compared with Kaplan-Meier analysis. EVF was an independent risk factor for mortality in NSTEMI patients below (HR: 2.4) and above the age of 70 (HR: 2.1). Mortality rates between the two age groups of NSTEMI patients with EVF did not differ significantly: 30-day mortality was 24% vs 40% (*p*=0.2709) and 1-year mortality was 39% vs 55% (*p*=0.2085). Additional mortality after 30 days to 1 year was 15% vs 14.6% (*p*=0.9728). Clinical characteristics of patients with EVF differed significantly from those without in both age groups. EVF after revascularization—within 48 h—had 11.2 OR for 30-day mortality above the age of 70. EVF in NSTEMI was an independent risk factor for mortality in both age groups. Invasive management and revascularization of NSTEMI patients with EVF is highly recommended. Closer follow-up and selection of patients (independent of age) for ICD implantation in the critical first month is essential.

## Introduction

Ventricular arrhythmias are potentially lethal complications of acute coronary syndromes. According to the VALLIANT Trial, the risk of sudden cardiac death is the highest within 1 month after the infarction [1]. Early primary ventricular fibrillation (EVF) occurs within 48–72 h after the symptoms’ onset and it is independent of the reoccurring ischemia and heart failure. In daily clinical practice, the prognosis of patients surviving EVF is not clear.

However, the GISSI-2 Trial showed the relevance of EVF as an independent predictor for in-hospital mortality [2]. Results regarding the risk factors and the effect of EVF on the short- and long-term prognoses in ST-segment elevation myocardial infarction (STEMI) patients have been controversial. EVF is a predictor for both 30-day and 1-year mortality rates in STEMI patients treated with primary percutaneous coronary intervention [3]. In contrast, another study suggested that EVF was associated with higher in-hospital mortality but did not affect the long-term prognosis [4]. Other earlier studies also found non-significant impact of EVF on prognosis [5], and recent studies did not confirm these results [6]. Medina-Rodriguez et al. found that EVF before intensive care unit admission was an independent predictor of in-hospital mortality in a cohort of patients in whom fibrinolysis was the main method of revascularization therapy [7]. A similar prognostic impact in patients treated with percutaneous coronary intervention (PCI) was not present in that study, suggesting that PCI has a long-term therapeutic benefit in EVF patients. In a large unselected population of STEMI patients treated with PPCI, ventricular fibrillation during the first 48 h after STEMI was associated with increased in-hospital mortality but no influence on the long-term prognosis for surviving patients was established [8]. In contrast, Kosmidou et al. have reported that ventricular arrhythmias occurring before coronary angiography and revascularization in patients with STEMI were strongly associated with an increased 3-year likelihood of death and stent thrombosis [6].

Jabbari et al. investigated the independent risk factors that contribute to the occurrence of ventricular fibrillation (VF) before PPCI in STEMI patients. They found that traditional coronary artery disease (CAD) risk factors such as diabetes, hypertension, and hypercholesterinemia did not predict risk while higher age, family history of sudden cardiac death, use of statins, and higher alcohol intake were independent risk factors [9]. The extent of the CAD also contributed to higher mortality. Larger studies found that EVF is associated with the final infarct size [10, 11]. However, the findings of Gheeraer et al. contradicted these results, reporting that the region at risk and the site of the occlusion are not independent risk factors for out-of-hospital VF [12]. Literature data suggests that in STEMI, EVF is an independent risk factor for in-hospital and short-term mortality, and its effect on long-term mortality is unclear.

Many previous studies have investigated the risk factors contributing to EVF and the prognosis of EVF in unselected myocardial infarction population. A high proportion of acute coronary syndrome cases is NSTEMI. In NSTEMI patients, the prognosis of EVF and factors influencing the prognosis are less clarified. NSTEMI patients are more likely to exhibit complicated cases, have more comorbidities, and have higher mortality rates in general. In addition, mean age in NSTEMI is higher, and as a result of all these factors, it is essential to evaluate this patient population. Since NSTEMI prevalence increases with age, the importance of evaluating this patient population is particularly well justified. Current guidelines have few instructions about the management of NSTEMI patients with EVF, largely due to a lack of study evaluation in this population, especially in patients at higher age.

To help combat this lack of data about the prognosis and management of NSTEMI patients surviving EVF, the present study using our large database (~ 12,000 patients) has been undertaken. Our goal was to investigate the risk factors contributing to early VF in the elderly and identify factors influencing the prognosis of NSTEMI subjects using a retrospective study design.

## Methods

### Study population and data collection

A total of 11,582 patients with acute coronary syndrome have been revascularized between 2005 and 2013 at our institution. These consecutive patients were enrolled in the Városmajor Myocardial Infarction Registry (VMAJOR-MI Registry), in which all the available demographic data and clinical data are summarized. Demographic data include gender, date of birth, date of admission, and date of death. Clinical patient data include laboratory findings (troponin T, CK-MB, creatinine, glucose, cholesterol, LDL-cholesterol), type of infarction (STEMI, NSTEMI), results from echocardiography, left ventricle ejection fraction (LV-EF), and coronary angiography. The initiating acute event has been characterized by the following factors: complicated by EVF, cardiogenic shock, on-site resuscitation, heart failure, invasive respiratory treatment. Data from EVF-positive patients was supplemented by information on laboratory parameters such as potassium levels, white blood cell (WBC) count, and C-reactive protein (CRP) levels, as well as by information on detailed coronary status such as the number of vessels affected, and the number of vessels treated by PCI.

**Fig. 1:**
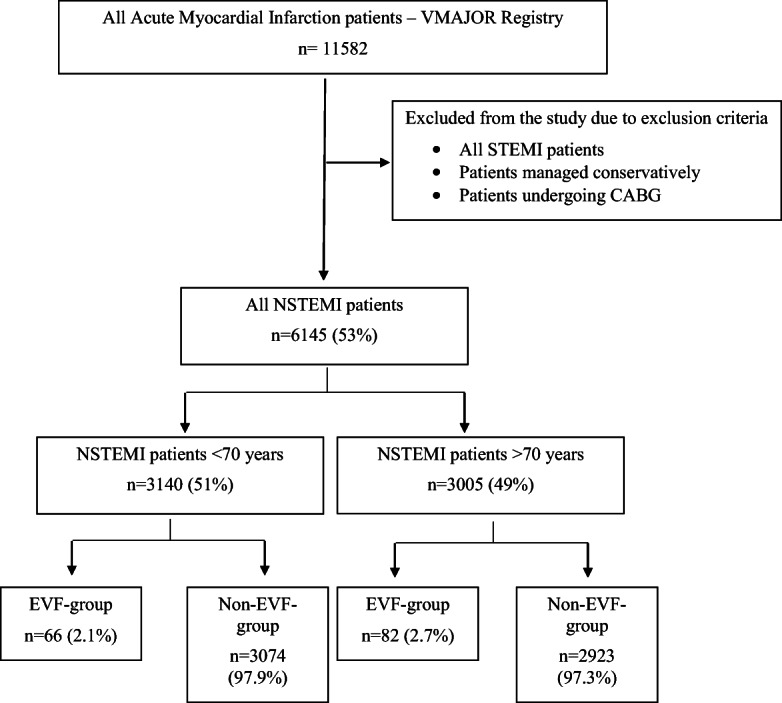
Process of patient enrolment

From this detailed VMAJOR-Registry, we enrolled only patients having NSTEMI. Patients with ST-segment elevation myocardial infarction were excluded from our analysis. We divided patients into two groups based on whether or not their myocardial infarction led to EVF. Patients were further grouped based on age (above or below the age of 70 years). Figure 1 describes the enrolment and grouping process in detail.

Diagnosis of NSTEMI was made based on typical symptoms such as chest discomfort, upper extremity discomfort, dyspnea, fatigue, and the elevation of necrosis markers. EVF was defined as ventricular fibrillation requiring defibrillation in the first 48 h after AMI. Patients in the EVF group included those who suffered VF before or after revascularization as long as it was within 48 h. Other types of ventricular arrhythmias, such as ventricular tachycardia, were not examined.

Among NSTEMI patients, only patients undergoing coronary angiography, defined as percutaneous coronary intervention (PCI) in the first 12 h of symptom onset, were included in the study [13]. Patients undergoing coronary artery bypass grafting surgery or who were managed conservatively were not eligible for the study. Coronary stenosis was evaluated from multiplane projections and a luminal diameter reduction of >50% was considered significant. The study protocol conformed to the ethical guidelines of the 1975 Declaration of Helsinki and was approved in advance by the locally appointed ethics committee (30088-2/2014/EKU). The primary outcome of the study was all-cause mortality. The National Health Care Institute provided accurate details on the above endpoint with occurrence dates.

### Statistical methods

Analysis was performed using Statistica 13.2 software and MedCalc statistical software. Continuous variables were expressed as mean and standard deviation (mean ± St. D); categorical variables were summarized as the sample size (*n*) and frequencies. Student’s *t*-test was used for comparison of normally distributed data and Mann-Whitney *U*-test with non-normal distribution. Categorical variables were compared using chi-square test.

Cox proportional hazard model was used to identify whether EVF was an independent risk factor for mortality. The model included the general risk factors such as age, gender, diabetes mellitus, left ventricle function, severity of acute event—heart failure, cardiogenic shock, invasive respiratory treatment—and EVF.

Cox regression analysis was performed in order to identify clinical characteristics associated with mortality. The model included all available risk factors of acute coronary syndromes and ventricular fibrillation such as gender, age, body mass index (BMI), diabetes mellitus, LV-EF, complications of the acute event (on-site CPR, cardiogenic shock, heart failure), coronary angiographic results (coronary status—vessels affected, PCI results—stent implantation on how many vessels), and the time of the VF (before, during, or after the revascularization but within 48 h). Hazard ratios (HR) with corresponding 95% confidence intervals (CI) were calculated using Cox proportional hazard model. Survival time of the different patient groups was compared using Kaplan-Meier survival analysis. All statistical analysis was two-tailed; the level of significance was *p*<0.05.

## Results

The clinical characteristics of the 3140 NSTEMI patients below the age of 70 are presented in Table 1. Significant differences were found between EVF-positive and EVF-negative cases. EVF-positive NSTEMI patients were more likely to have poor left ventricle function (LV-EF <40%) (39.22% vs 14.95%), larger infarct size characterized by higher troponin (2073.9 ng/L vs 902.3 ng/L), and higher CK-MB (158.7 U/L vs 74.8 U/L), and were more likely to have diabetes (55.56% vs 30.34%). They suffered more severe infarction with more complications such as cardiogenic shock (18.18% vs 2.15%) and increased need for invasive respiratory treatment (43.94% vs 5.6%). Given these significant differences, it is not surprising that EVF-positive patients also had higher mortality rates than control patients. 30-day mortality was 24% vs 4.6% and 1-year mortality was 39% vs 10.6% in EVF vs. non-EVF patients <70, respectively. However, additional mortality (mortality between 30 days and 1 year) did not differ significantly.

**Table 1:** Clinical characteristics of NSTEMI patients, differences between EVF-positive and EVF-negative patients

Characteristics of patients < 70 years			
	With EVF	Without EVF	*p*-value
Gender, male (%)	66.67% (44/66)	68.58% (2108/3074)	0.7871
Mean BMI (kg/m^2^)	29.2±6.4	28.75±5.5	0.564
LV-EF <40% (%)	**39.22% (20/51)**	**14.95% (367/2455)**	**0.0041**
Serum creatinine >100 umol/L (%)	**39.68% (25/63)**	**20.56% (588/2860)**	**0.0223**
Mean troponin T (ng/L)	**2073.9±3219.1**	**902.3±1992.2**	**0.0006**
Mean CK-MB (U/L)	**158.7±195.2**	**74.8±94.3**	**<0.0001**
DM (%)	**55.56% (35/63)**	**30.34% (850/2802)**	**0.0016**
Mean cholesterine (mmol/L)	4.65±1.5	4.79±1.4	0.4649
Mean LDL-cholesterol (mmol/L)	2.88±1.33	3.05±1.3	0.3811
Heart failure (%)	27.27% (18/66)	14.38% (442/3074)	0.132
Cardiogenic shock (%)	**18.18% (12/66)**	**2.15% (66/3074)**	**0.0149**
Resuscitation (%)	**31.82% (21/66)**	**0.91% (28/3074)**	**0.0021**
Invasive respiratory treatment (%)	**43.94% (29/66)**	**5.6% (172/3074)**	**<0.0001**
Mean survival (days)	**1587.9±1465.67**	**1924.5±1079.8**	**0.013**
30-day mortality (%)	**24.24% (16/66)**	**4.59% (141/3074)**	**0.0027**
1-year mortality (%)	**39.39% (26/66)**	**10.61% (326/3074)**	**<0.0001**
Mortality between 30 days and 1 year (%)	15.15% (10/66)	6.02% (185/3074)	0.2536

*p-value: difference between EVF-positive and EVF-negative NSTEMI patients. Statistical significant differences are highlighted in bold

Abbreviations: *BMI*, body mass index; *LV-EF*, left ventricle ejection fraction; *DM*, diabetes mellitus

Table 2 shows the differences between EVF and non-EVF groups in patients above the age of 70 years. In the older age group, similarly to the younger patient group, subjects surviving EVF were more likely to have reduced left ventricle ejection fraction (44.4% vs 22.8%) and diabetes (53% vs 37%). They also had more severe complications after the acute event including cardiogenic shock (18% vs 4%) and the need for invasive respiratory treatment (56% vs 9%). As seen in the <70 patient group, the patients aged >70 also exhibited increased 30-day and 1-year mortality in the EVF group vs non-EVF group (40% vs 10% for 30 days and 55% vs 28% for 1 year).

**Table 2:** Clinical characteristics of NSTEMI patients above the age of 70 years, differences between EVF-positive and EVF-negative patients

Characteristics of patients > 70 years			
	With EVF	Without EVF	*p*-value
Gender, male (%)	64.63% (53/82)	52.96% (1548/2923)	0.094
Mean BMI (kg/m^2^)	26.6±4.4	27.1±4.8	0,4132
LV-EF <40% (%)	**44.44% (32/72)**	**22.77% (529/2323)**	**0.0053**
Serum creatinine >100 umol/L (%)	58.23% (46/79)	44.04% (1205/2736)	0.0574
Mean troponin T (ng/L)	1235.7±1695.8	841.1±1638.1	0.1232
Mean CK-MB (U/L)	**103.5±94.8**	**77.3±94**	**0.0311**
DM (%)	**53.16% (42/79)**	**37.01% (989/2672)**	**0.0344**
Mean cholesterol (mmol/L)	**3.9±1.4**	**4.3±1,3**	**0.0167**
Mean LDL-cholesterol (mmol/L)	2.3±1.3	2.6±1.2	0.0666
Heart failure (%)	40.24% (33/82)	26.27% (768/2923)	0.0762
Cardiogenic shock (%)	**18.29% (15/82)**	**4.38% (128/2923)**	**0.0297**
Resuscitation (%)	**29.27% (24/82)**	**0.68% (20/2923)**	**0.0105**
Invasive respiratory treatment (%)	**56.1% (46/82)**	**8.93% (261/2923)**	**<0.0001**
Mean survival (days)	**747.3±1026.9** ***n*=82**	**1255.8±1032.3**	**<0.0001**
30-day mortality (%)	**40.24% (33/82)**	**10.20% (298/2923)**	**<0.0001**
1-year mortality (%)	**54.88% (45/82)**	**28.26% (826/2923)**	**0.0001**
Mortality between 30 days and 1 year (%)	14.63% (12/82)	18.06% (528/2923)	0.7597

*p*-value: difference between EVF-positive and EVF-negative NSTEMI patients. Statistical significant differences are highlighted in bold

Abbreviations: *BMI*, body mass index; *LV-EF*, left ventricle ejection fraction; *DM*, diabetes mellitus

When we compared mortality rates for NSTEMI patients surviving EVF in patients below 70 years vs above 70 years, no significant difference has been found in 30-day mortality (24% vs 40% *p*=0.2709), in 1-year mortality (39% vs 55% *p*=0.2085), or in mortality between 30 days and 1 year (15% vs 14.6% *p*=0.9728).

**Fig. 2:**
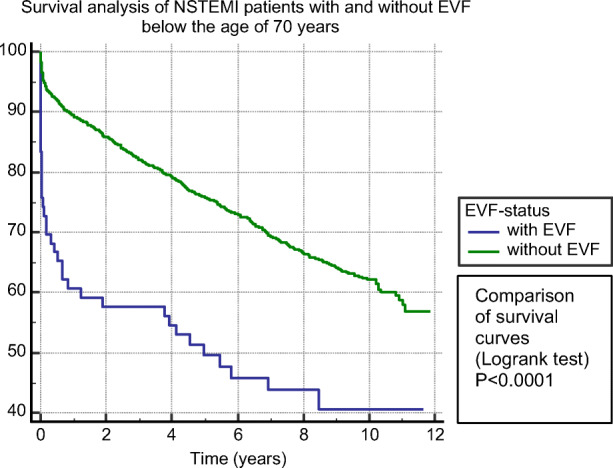
Survival analysis of EVF-positive compared to EVF-negative NSTEMI patients below the age of 70 years. Abbreviations: NSTEMI, non-ST-segment elevation myocardial infarction; EVF, early ventricular fibrillation

**Fig. 3:**
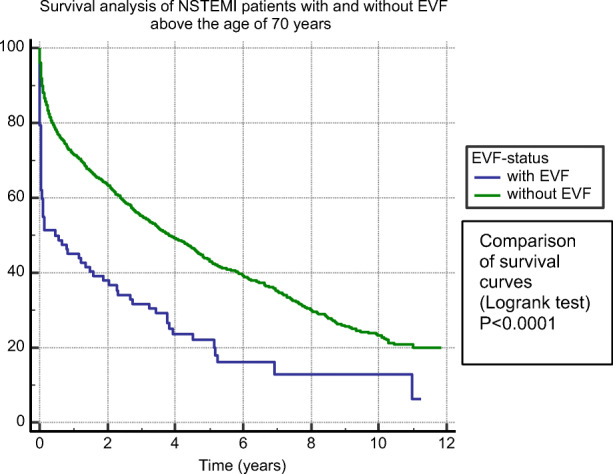
Survival analysis of EVF-positive compared to EVF-negative NSTEMI patients above the age of 70 years. Abbreviations: NSTEMI, non-ST-segment elevation myocardial infarction; EVF, early ventricular fibrillation

**Fig. 4:**
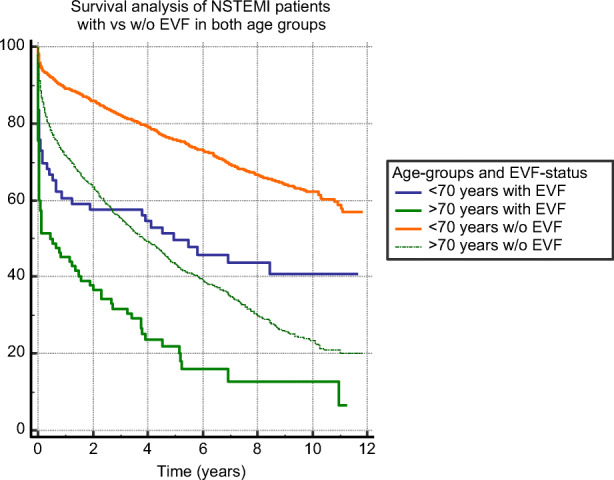
Survival analysis of EVF-positive compared to EVF-negative NSTEMI patients in both age groups. Abbreviations: NSTEMI, non-ST-segment elevation myocardial infarction; EVF, early ventricular fibrillation

EVF patients at younger (Fig. 2), as well as at older age, >70 years (Fig. 3), had significantly (*p*<0.0001) lower survival probability compared to non-EVF ones. Figure 4 shows survival probability in the 4 patient groups (based on age and EVF).

Cox regression analysis showed that in patients <70, EVF is an independent risk factor for all mortality (HR: 2.38) (Table 3), in addition to other factors such as diabetes mellitus (HR: 2.02), heart failure (HR: 3.66), cardiogenic shock (HR: 8.99), and invasive respiratory treatment (HR: 5.4). Similarly, in patients above the age of 70 years, EVF is also an independent risk factor for mortality (HR: 2.1) as well as diabetes mellitus (HR: 1.5), heart failure (HR: 2.4), cardiogenic shock (HR: 4.85), and invasive respiratory treatment (HR: 3.2) (Table 4).

**Table 3:** Cox regression analysis of factors influencing mortality in NSTEMI patients below the age of 70 years

Covariate	HR	95% CI of HR	*p*-value
LV-EF (%)	0.9549	0.9505 to 0.9593	<0.0001
Diabetes mellitus	2.0263	1.7685 to 2.3216	<0.0001
Cardiogenic shock	8.9915	6.9951 to 11.5577	<0.0001
Invasive respiratory treatment	5.4124	4.5299 to 6.4668	<0.0001
Heart failure	3.6573	3.1696 to 4.2201	<0.0001
Early ventricular fibrillation	2.3813	1.7133 to 3.3097	<0.0001

Abbreviations: *LV-EF*, left ventricle ejection fraction

**Table 4:** Cox regression analysis of factors influencing mortality in NSTEMI patients above the age of 70 years

Covariate	HR	95% CI of HR	*p*-value
BMI	0.9804	0.9693 to 0.9917	0.0007
LV-EF (%)	0.967	0.9633 to 0.9706	<0.0001
Diabetes mellitus	1.5217	1.3822 to 1.6754	<0.0001
Cardiogenic shock	4.8505	4.0652 to 5.7875	<0.0001
Invasive respiratory treatment	3.244	2.8442 to 3.7001	<0.0001
Heart failure	2.3965	2.1761 to 2.6392	<0.0001
Early ventricular fibrillation	2.1383	1.6780 to 2.7249	<0.0001

Abbreviations: *BMI*, body mass index; *LV-EF*, left ventricle ejection fraction

Seeing that EVF is an independent risk for mortality in NSTEMI in both age groups, we furtherly evaluated the risk factors for mortality in the NSTEMI with EVF patient group. Factors influencing mortality in NSTEMI patients surviving EVF are presented in Table 5. In patients below the age of 70 years, these factors include diabetes mellitus (HR: 1.9), cardiogenic shock (HR: 6.1), heart failure (HR: 2.65), and CPR (HR: 2.5). However, at higher age (above 70 years), the only factor influencing mortality was cardiogenic shock (HR: 2.3). The extent of the coronary artery disease did not affect mortality in either age group.

**Table 5:** Cox regression analysis of factors influencing mortality in NSTEMI patients surviving EVF

NSTEMI patients surviving EVF below the age of 70				NSTEMI patients surviving EVF above the age of 70			
Covariate	HR	95% CI of HR	*p*-value	Covariate	HR	95% CI of HR	*p*-value
LV-EF (%)	0.9506	0.9226 to 0.9795	0.0009	LV-EF (%)	0.9716	0.9503 to 0.9934	0,011
Diabetes mellitus	1.9262	1.2884 to 2.8797	0.0014	Diabetes mellitus	1.467	0.8977 to 2.3974	0.1262
Cardiogenic shock	6.1416	2.9553 to 12.7635	<0.0001	Cardiogenic shock	2.3205	1.2655 to 4.2551	0.0065
Heart failure	2.6468	1.3432 to 5.2154	0.0049	Heart failure	1.3306	0.8119 to 2.1807	0.2571
CPR	2.5042	1.3009 to 4.8206	0.006	CPR	1.0754	0.6297 to 1.8368	0.7901
2-vessel disease*	1.2863	0.5665 to 2.9207	0.5473	2-vessel disease*	1.1272	0.6895 to 1.8427	0.6330
3-vessel disease*	2.1285	0.8956 to 5.0583	0.0872	3-vessel disease*	1.7589	0.9067 to 3.4119	0.0948

Abbreviations: *LV-EF*, left ventricle ejection fraction; *CPR*, cardiopulmonary resuscitation

*Compared to 1-vessel disease based on coronary angiographic results

To help understand whether the timing of EVF, with respect to the timing of the coronary revascularization, had an effect on prognosis, we asked whether mortality was different in patients who experienced EVF either before or after PCI. In most cases in the <70 age group, EVF developed before revascularization (75%, 51/68), but the timing had no influence on either the short- or long-term mortality (Table 6). In the higher age group (>70 years), most EVF also developed before revascularization (74%, 61/82). In contrast to the younger group, in patients >70 years, EVF that occurs after revascularization was associated with a higher risk of 30-day mortality (OR 11.2), although 1-year mortality was not significantly different (Table 7).

**Table 6:** The effect of the EVF’s occurrence on the 30-day and 1-year mortality in all NSTEMI patients

	Total	Dead	%	*p*	OR	OR 95% CI
30-day mortality in patients below the age of 70						
VF during rev.*	12	1	8.33	*-*		
VF before rev.*	51	13	25.49	0.1984	0.2657	0.0312–2.2626
VF after rev.* <48 h	5	2	40	0.1186	7.3	0.4836–111.19
1-year mortality in patients below the age of 70						
VF during rev.*	12	2	16.67	*-*		
VF before rev.*	51	22	43.14	0.0893	0.2636	0.0524–1.327
VF after rev. <48 h*	5	2	40	0.3014	3.33	0.319–34.83

*rev., revascularization

**Table 7:** The effect of the EVF’s occurrence on the 30-day and 1-year mortality in NSTEMI patients above 70 years

	Total	Dead	%	*p*	OR	OR 95% CI
30-day mortality above the age of 70						
VF during rev.*	9	1	11.11%	*-*		
VF before rev.*	61	25	40.98%	0.0834	0.18	0.021–1.53
VF after rev.* <48 h	**12**	**7**	**58.33%**	**0.027**	**11.2**	**1.04–120.4**
1-year mortality above the age of 70						
VF during rev.*	9	4	44.44%	*-*		
VF before rev.*	61	32	52.46%	0.653	0.725	0.177–2.962
VF after rev. <48 h*	12	9	75%	0.153	3.75	0.587–23.94

*rev., revascularization

## Discussion

According to the literature, the incidence of ventricular arrhythmias including EVF in the acute phase of MI is approximately 2–8% [7, 14], which is consistent with data in our patient group resented here (2.4%). Despite the fact that the incidence of ventricular arrhythmias is higher in STEMI than in NSTEMI (10% vs 2.1%) respectively [15], mortality rates in the EVF patient group are significantly higher regardless of the infarction type versus non-EVF patients. However, the FAST-MI program discovered that 6-month mortality has decreased over the past 20 years [16]. Since 2010, mortality in STEMI patients has continued to decline; however, mortality in NSTEMI patients has remained stable [16], highlighting the need for further investigation into factors that affect NSTEMI mortality. In a small group of invasively treated NSTEMI patients, Gupta et al. investigated the incidence of and predictors for malignant arrhythmias [17]. In their population, VF occurred in 7.6% of the patients, a much higher fraction than in our study in which 2.4% (151/6179) experienced EVF. Similar to our study, they also reported that 30-day mortality was significantly higher in patients with vs. without VF (38% vs 3%), and their 30-day mortality rate among EVF patients was comparable to what we observed (33%). However, their EVF-negative patients had higher 30-day mortality compared to our results despite using invasive therapy. Similarly, Al-Khatib et al. also reported increased 30-day and 6-month mortality in spite of using effective therapy [15]. They found that in-hospital VF and VT were independently associated with 30-day and 6-month mortality even after excluding patients with heart failure and cardiogenic shock and those who died within 24 h [15]. The MERLIN-TIMI 36 Trial also highlighted the significance of non-sustained VT in NSTEMI. Although non-sustained VT is common after NSTEMI, short episodes of VT are independently associated with a higher risk of sudden cardiac death [18].

In the intervention era (2000–2012), the number of patients who receive coronary angiography and PCI after VT/VF has increased, resulting in a higher survival rate—survival in all acute myocardial infarction has risen from 46.9 to 60.1%, in STEMI survival has risen from 59.2 to 74.3%, and in NSTEMI survival has risen from 43.3 to 56.8% [19]. In spite of clear evidence showing that coronary angiography and PCI increase survival, in daily practice, some proportion of patients, mostly NSTEMI, do not undergo revascularization. At our high-volume cardiology institute with an invasive approach, STEMI and NSTEMI patients are treated invasively. Yet, in spite of this invasive strategy, here, we report that NSTEMI patients surviving EVF still have higher short- and long-term mortality rates compared to those without EVF regardless of whether they are above or below 70 years of age. Kaplan-Meier analysis (Fig. 4) showed that in the first 3 years after the acute myocardial infarction, younger patients with EVF had worse survival probability than those older ones without EVF. However, past the 3 years’ mark, age becomes more important regards life expectancy. This finding supports the fact that age is one of the main determining factors of survival. Besides the clinical fact that older patients are more likely to have numerous comorbidities, cellular and molecular mechanisms may contribute to increased mortality in older adults, e.g., impaired cellular stress and age-related oxidative stress. Studies confirm this idea that hypoxia, oxidative stress, worsens the prognosis of cardiovascular patients. Trimetazidine was found to be a cytoprotective agent [20] which improves the quality of life and left ventricle function in elderly patients with ischemic heart disease [21, 22]. Importantly, our analysis demonstrated that in addition to other well-characterized comorbidities, EVF is also an independent risk factor for mortality in both age groups. This is consistent with other studies showing that patients with EVF (compared to non-EVF patients) have more frequently reduced LV-EF and triple-vessel coronary artery disease (CAD) resulting in higher 30-day mortality [23]. Interestingly, the prognosis had no correlation with the extent of the coronary artery disease either at lower or at higher age in NSTEMI patients surviving EVF. In NSTEMI patients with EVF at younger age, several factors had an influence on mortality, such as LV-EF, diabetes mellitus, cardiogenic shock, heart failure, and on-site resuscitation. In contrast to that at higher age, the only factor was a cardiogenic shock.

One interesting finding of our study was the timing of EVF (with respect to the timing of intervention) impacted the prognosis in older but no younger NSTEMI patients. The impact of timing on outcomes has also been examined by others. For example, according to Jabbari et al., there is no difference in the 30-day mortality in STEMI patients depending on VF before or during PCI [24].

In general, NSTEMI patients have worse prognosis than STEMI patients, and cases that are complicated with EVF have even poorer outcomes. The mortality risk was the highest within the first 30 days; we found that 40% of the aged patients died within the first month. There are only a few differences in clinical factors influencing who will develop EVF in the two age groups. The fact that EVF develops based on acute myocardial ischemia is not surprising. Our results suggest that this pathomechanism is the same at higher age. Invasive management of NSTEMI patients is essential, and even with invasive management, EVF was an independent risk factor for mortality. These findings suggest that closer follow-up—using telemedicine in the aged patient population with decreased mobility—in the critical first 30 days is essential. It is important to select patients, independently from age, who would benefit from an early implantable cardioverter-defibrillator implantation before discharge. With more outpatient visits, more precise medication setup for secondary prevention would be beneficial independently from age.

### Limitation section

The present study was a single-center retrospective observational study with limited available data. Factors affecting the prognosis such as ICD implantations, medication intake, and compliance could not be investigated.
